# Demosponge (Porifera) mitogenomes reflect latitudinal structuration, reveal intraspecific architecture variation, and confirm mitochondrial intron splicing

**DOI:** 10.1186/s13062-026-00913-1

**Published:** 2026-07-28

**Authors:** Carles Galià-Camps, Carlota Gracia-Sancha, Paco Cárdenas, Joana R. Xavier, Ana Riesgo, Sergi Taboada

**Affiliations:** 1 Department of Biodiversity and Evolutionary Biology, Museo Nacional de Ciencias Naturales (MNCN-CSIC), Calle José Gutiérrez Abascal 2, Madrid, 28006 Spain; 2 Sociedad de Amigos del Museo Nacional de Ciencias Naturales de Madrid (SAM), Calle José Gutiérrez Abascal 2, Madrid, 28006 Spain; 3 Museum of Evolution, Uppsala University, Norbyvägen 16, Uppsala, 752 36 Sweden; 4 CIIMAR/CIMAR LA, Interdisciplinary Centre of Marine and Environmental Research, University of Porto, Terminal de Cruzeiros do Porto de Leixões, Matosinhos, 4450-208 Portugal; 5 Department of Natural History, University Museum of Bergen, University of Bergen, Bergen, Norway

**Keywords:** WGS, RNA-seq, Mitogenome evolution, Insertion - deletion, Differential transcriptome coverage, Mitochondrial annotation

## Abstract

**Background:**

Mitochondrial markers have historically been underused in demosponge genetic studies due to their low intra- and interspecific variability. Consequently, demosponge complete mitogenomes remain largely unexplored, even though they provide maternally inherited information that complements nuclear markers. Here, we assembled the mitochondrial genomes of 28 specimens of the ground-forming demosponge *Phakellia ventilabrum* across its North Atlantic distribution range to characterize them and evaluate their performance compared to already published results using restriction-site-associated DNA sequencing (RADseq) data.

**Results:**

Our assemblies revealed two region-specific mitogenomes with divergent architectures and differential copy numbers. Moreover, we identified two mitochondrial introns: one in the *16S* gene and another in the *cox1* gene. The *cox1* intron could be assigned to a group II intron, whereas the *16S* intron could not be confidently assigned either to group I or group II introns. Nevertheless, BLAST results suggested horizontal gene transfer events from yeasts in both cases. The integration of RNA-seq data showed that intronic expression was significantly lower than that of exons in both the *16S* and *cox1* genes, confirming intron boundaries and providing strong evidence for intron splicing functionality. The genetic differentiation pattern recovered with mitochondrial markers is consistent with previous nuclear data, suggesting a differentiation between southern and northern localities. We further revealed two sympatric mitogenomic lineages in the north. Significantly, *nad2* mirrored the same lineages recovered using complete mitogenomes.

**Conclusions:**

This study demonstrates the value of mitogenomes for the robust evaluation of lineage differentiation in demosponges and provides new approaches for research on mitochondrial intron acquisition.

**Supplementary Information:**

The online version contains supplementary material available at https://doi.org/10.1186/s13062-026-00913-1.

## Background

Deep-sea sponge grounds create structurally complex habitats that support and enhance local biodiversity [1]. While these ecosystems are known to occur globally, they form large, dense aggregations primarily at latitudes greater than 45º north and south [2–4]. The ecology and distribution of sponge grounds in the North Atlantic have been relatively well studied as these habitats are home to several commercially important deep-sea fishes (e.g. [5–8]). Productivity in these regions is exceptionally high, and these areas have become a key target of present-day fishing expeditions [1, 9, 10]. As a consequence of fishing exploitation, the health of many sponge-ground-forming species is severely compromised, such as *Geodia* spp. in the Barents Sea [11] or *Polymastia thielei* Koltun, 1964 [12] in the Arctic Sea [13]. These emerging pressures pose a major threat to global ecosystem balance, as engineering sponge species are especially vulnerable to external stressors due to their extremely slow recovery rates [13]. Consequently, the degradation of sponge grounds triggers cascading biodiversity loss, leading to mass mortality events and the potential extinction of species that depend on these remote yet essential habitats [1, 14]. Because of the bio-socio-economic relevance of sponge grounds, considerable efforts are being made to understand the connectivity among deep-sea sponge ground communities [15–20]. As commonly observed for other deep-sea organisms, deep-water sponges exhibit remarkable latitudinal connectivity over hundreds or even thousands of km when studied at similar depths [18, 19, 21–23]. However, genetic structure in the deep sea is typically observed when investigating organisms occurring across wide bathymetric ranges, resulting in isolation of lineages [21, 23]. This isolation by depth has been reported in several deep-water marine invertebrates such as the coral *Montastraea cavernosa* (Linnaeus, 1767) [24, 25] and has been reported for the first time in sponges in the species *Geodia hentscheli* [23].

Understanding the connectivity patterns of deep-sea species, particularly key ecosystem engineers such as sponges, is central to designing efficient marine protected areas aiming to alleviate the impacts of climate change and seabed mining, among others, while preserving the welfare of oceans [26]. However, the deep sea remains highly understudied, hampering efforts to evaluate and mitigate anthropogenic impacts that threaten the integrity of these fragile ecosystems [26]. Closing this gap is imperative for sustainable management and the long-term preservation of ocean health. Most studies aiming to establish the genetic connectivity in sponges have used traditional markers such as the cytochrome oxidase I (*cox1*) [27, 28] or microsatellites [22, 27, 29–31], and only a few have further elaborated these findings using a few thousand nuclear markers through restriction-site-associated DNA sequencing (RADseq) [18, 19, 23, 32, 33]. RADseq methods produce large numbers of single nucleotide polymorphisms (SNPs), randomly distributed across the genome of the species under study [34, 35]. However, in sponges, these efforts to better sample variation across the nuclear genome have often lacked a reference genome, thereby limiting the technique’s potential. With the advent of next-generation sequencing (NGS) techniques, other more efficient approaches have recently emerged. Such is the case of whole-genome sequencing (WGS), which significantly enhances the detection of genomic lineages and demographic histories at finer spatial and temporal scales, which can be even magnified with the aid of reference genomes [36–38]. In addition to providing an almost complete and continuous set of nuclear genetic data compared to RADseq approaches, WGS also recovers organellar reads that can subsequently be assembled to produce complete mitochondrial genomes (e.g. [39]). Mitochondrial genomes (from now on referred to as mitogenomes) are relatively easy to retrieve due to the high mitochondrial copy number, understood as the number of mitochondria per cell of the target species, resulting in much higher (tens of fold-times) coverage for the organellar DNA compared to nuclear DNA [40]. Interestingly, compared with traditional markers, the study of these mitogenomes has produced valuable insights into phylogenetics (e.g. phylogeny of demosponges), evolution (e.g. extreme molluscan mitogenome variability), and population structure (e.g. connectivity of the Alaskan red king crab) [39–41], among others. This maternally inherited genetic information has also been essential for addressing complex evolutionary scenarios, such as lineage-specific rearrangements [38], gene gain-loss events [42, 43], presence of NUMTs and demographic trends [44], as well as accelerated evolutionary rates [45].

Mitochondrial introns are a rare feature in eukaryotes. Nevertheless, the presence of group I introns (whose translocation is mediated by endonucleases) and group II introns (whose translocation is mediated by reverse transcriptases) in mitogenomes has been sporadically reported in demosponges and homoscleromorphs [41, 46–54]. A remarkable study on this issue targeted *Acanthella acuta* Schmidt, 1862 [55], in which the authors reported the potential presence of an intron in the 16S ribosomal gene and corroborated the presence of two group II introns in two protein-coding genes [47]. Due to the resemblance of all poriferan intronic sequences to fungi or placozoan species, these introns have been suggested to be acquired through horizontal gene transfer, mostly from symbionts [41, 46–48, 54, 56]. Despite these findings, the splicing function of these introns has not been tested, as experimental approaches and/or transcriptional profiles have not been carried out for validation.

Historically, mitochondrial markers have been underused in demosponge genetic studies [57]. Sponge mitochondrial markers exhibit low variability (typically a handful of polymorphisms), possibly related to mitochondrial repair mechanisms [58], which limits their utility in inferring connectivity patterns in most species [22, 32, 59–61]. In these cases, small fragments of mitochondrial genes are usually used, sometimes with moderate resolution [62] but generally rendering a very shallow resolution compared to nuclear markers [27, 63]. Complete mitogenomes have been explored in the context of genomic differentiation in only a few demosponges, such as the endangered *Cliona patera* [64] or *Dendrilla antarctica* [32], both showing very low variability among sampling sites. Within demosponges, mitogenomes of the subclasses Keratosa and Verongimorpha are notoriously conserved intra- and interspecifically, but those of Heteroscleromorpha are generally more variable [27, 65, 66]. In this context, the use of complete mitogenomes offers new opportunities to assess and complement population connectivity studies (e.g. [39]). Yet, the extent to which this holds for all demosponge groups remains unknown. The urgent need to understand connectivity within and across sponge grounds to inform conservation efforts justifies the need to investigate mitogenomes to provide valuable insights for assessing different sponge-ground-forming lineages.

To assess the applicability of mitogenomes for sponge genetic differentiation analysis, we used the demosponge *Phakellia ventilabrum* (Linnaeus, 1767) [25] (order Bubarida, family Bubaridae) as a study model. *Phakellia ventilabrum* is a ground-forming species abundant and widely distributed across the Northeast Atlantic and less common in the Mediterranean Sea. In the former, they can form dense aggregations on the continental shelf [67–69]. It occurs within a wide bathymetric range, commonly inhabiting depths around 500 m, although stable populations also thrive in the shallow waters of Roscoff at 10 m and as deep as 1,800 m in the Cantabrian Sea [70]. Recently, the genetic connectivity patterns of *P. ventilabrum* have been investigated by integrating genomic data (ddRADseq) with oceanographic modeling, providing a valuable comparative background for this species [19]. In their study, Taboada et al., (2023) identified a population comprising samples from the Cantabrian Sea (North of Spain) and Roscoff (France), isolated from another panmictic population encompassing sampling sites from the Rockall Bank (West of Scotland) to the north of Norway. In the latter case, the long-distance connectivity across the panmictic population (spanning ca. 2,000 km) was mainly explained by the presence of prevalent northwards oceanographic currents [19]. Despite the robust support from these combined methods, the absence of samples from peripheral regions across its distribution area and the absence of mitochondrial data prevented obtaining a complete picture of the connectivity patterns of *P. ventilabrum*. Addressing these gaps would provide a more comprehensive understanding of the species’ population structure and dispersal dynamics.

**Fig. 1:**
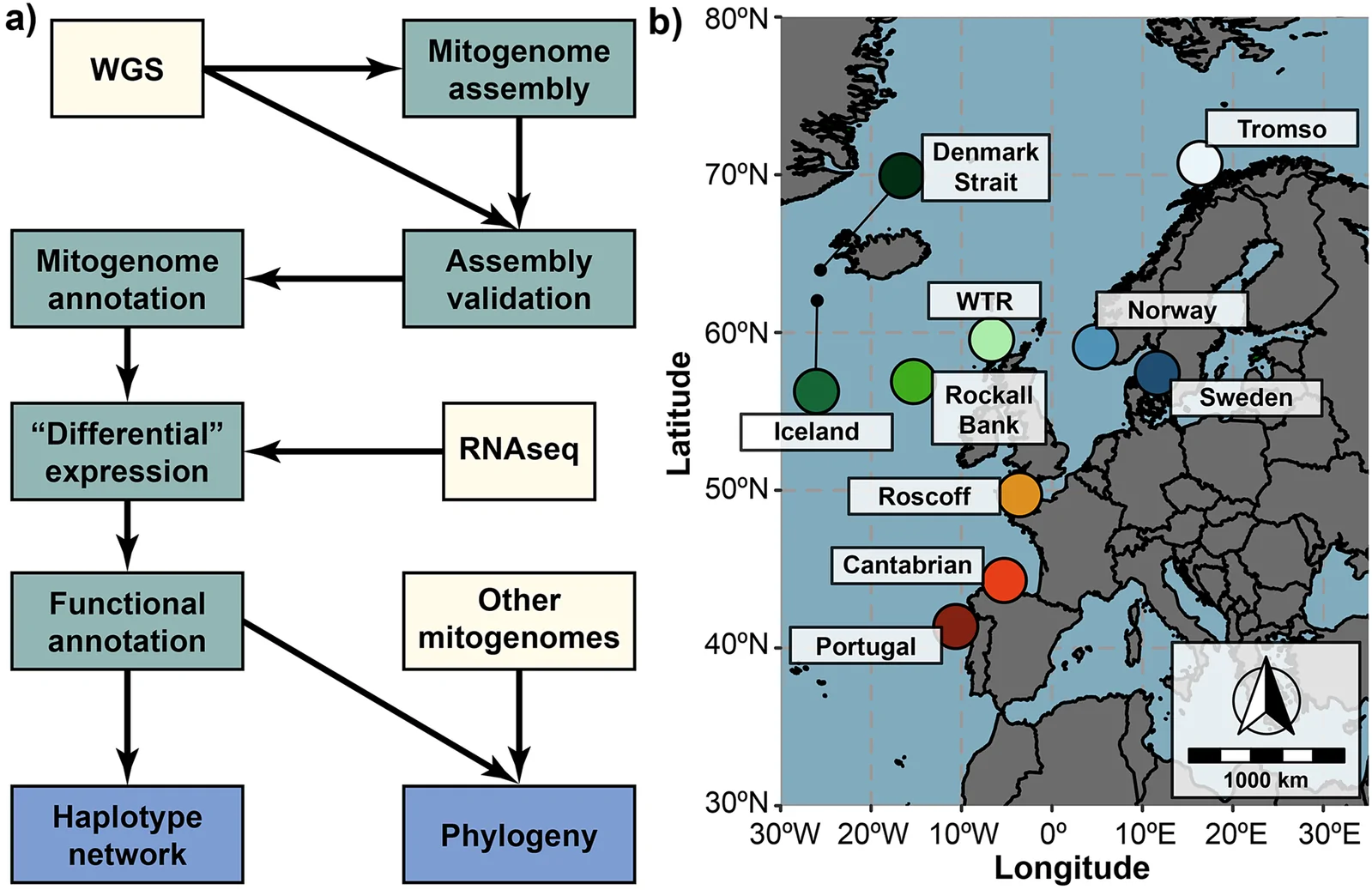
Study design and sampling locations. **a**) methodological flowchart followed in the present study. Yellow boxes represent the input data types, turquoise boxes indicate the steps for mitogenome assembly and annotation, and blue boxes highlight the analyses performed using the final versions of the mitogenome assemblies. **b**) map of the sampling locations. Note that the size of the circles does not correspond to the number of specimens. Note that sampling sites are named as in Taboada et al., (2023) for nomenclature consistency. WTR - Wyville Thomson Ridge

In the present study, we aim to unravel the mitochondrial genomic lineages of *P. ventilabrum* across the Northeast Atlantic, including samples from borderline localities in Iceland and Portugal. Specifically, we (I) use WGS data to assemble the mitogenome of 28 specimens, validate the assembled structures, and annotate them. Once assembled, we (II) evaluate diversity across different mitogenome architectures and retrieve statistics supporting their divergence. Afterward, we (III) combine mitochondrial assemblies with RNA-seq data to verify the presence of potential introns by analyzing differential expression patterns across genic regions (e.g. exon–intron boundaries). Finally, we (IV) explore the mitochondrial genomic lineages and their phylogenetic relationships (Fig. 1a).

## Material and methods

### Sampling, preservation, and morphological identification

Details on specimen collection and preservation can be found in Taboada et al. (2023) for all the sampling stations, as the specimens examined here correspond to those analyzed in that study (Table 1). Exceptions are the samples from Portugal, Sweden, and Iceland (Denmark Strait), which are novel to this study (Table 1). In summary, a total of 28 specimens of *P. ventilabrum* were collected using SCUBA diving, Remotely Operated Vehicles (ROVs), bottom trawls, trammel nets, and epibenthic sleds in 10 localities spanning approximately 3,600 km (Fig. 1, Table 1). Sampling depths ranged from ~30 m at Roscoff (NW France) to ~900 m at the Wyville Thomson Ridge (WTR, N Great Britain) (Table 1). Specimens were rinsed with clean seawater to remove sediment, identified to the genus or species level by sponge taxonomists, and photographed on board. For each specimen, tissue samples (1–9 cm^3^) were excised using sterile scissors, preserved in 96% ethanol, and stored at −20 °C, except for Swedish samples which were kept at room temperature. Ethanol was replaced daily for three days to ensure water removal and enhance DNA integrity.

**Table 1:** Sample metadata for the 28 specimens included in the study. We provide their sampling campaign ID, short ID, locality name, coordinates of the precise sampling site, collection date, method, and depth. Specimens from the Portugal, Sweden, and Iceland (Denmark Strait) sampling sites represent new material, while specimens from all other sites were previously evaluated using RADseq by Taboada et al. (2023)

Sample ID	Short ID	Locality	Latitude	Longitude	Collection date	Stations	Collection method	Collection Depth	mitochondrial haplogorup	Mitogenome accession number
CS_DR1_2_1	Cantabria_1	Cantabrian Sea	43º43’7.03’’ N	5º50’4.80’’ W	10–06-2017	DR1	Rock dredge	240	A	PX560193
CS_DR1_2_3	Cantabria_2	Cantabrian Sea	43º43’7.03’’ N	5º50’4.80’’ W	10–06-2017	DR1	Rock dredge	240	A	PX560194
CS_DR8_60	Cantabria_3	Cantabrian Sea	43º47’0.18’’ N	6º11’7.33’’ W	02–05-2010	DR8	Rock dredge	844	A	PX560191
Deep_094_02	Portugal_1	Portugal	41°25’0.12” N	9°34’59.88” W	30–04-2021	94	Trammel nets	58	A	PX560195
Deep_094_05	Portugal_2	Portugal	41°25’0.12” N	9°34’59.88” W	30–04-2021	94	Trammel nets	58	A	PX560196
Deep_094_06	Portugal_3	Portugal	41°25’0.12” N	9°34’59.88” W	30–04-2021	94	Trammel nets	58	A	PX560192
Dstrait_1	Strait_1	Iceland, Denmark strait	64°14’4.00’’ N	26°54’3.66’’ W	11–10-2016	A11-2016–522	Bottom trawl	354–435	B1	PX560197
Dstrait_2	Strait_2	Iceland, Denmark strait	64°14’4.00’’ N	26°54’3.66’’ W	11–10-2016	A11-2016–522	Bottom trawl	354–435	B1	PX560207
Dstrait_3	Strait_3	Iceland, Denmark strait	64°14’4.00’’ N	26°54’3.66’’ W	11–10-2016	A11-2016–522	Bottom trawl	354–435	B2	PX560206
ME85_1082_D	Iceland_1	Iceland	63°42’2.70’’ N	26°23’6.40’’ W	12–09-2011	ME872	Epibenthos sledge	705–711	B2	PX560214
ME85_1082_E	Iceland_2	Iceland	63°42’2.70’’ N	26°23’6.40’’ W	12–09-2011	ME872	Epibenthos sledge	705–711	B2	PX560201
ME85_1082_F	Iceland_3	Iceland	63°42’2.70’’ N	26°23’6.40’’ W	12–09-2011	ME872	Epibenthos sledge	705–711	B2	PX560215
NwK_KB_61_2	Norway_1	Norway Korsfjorden	59º58’8.79’’ N	5º22’4.37’’ E	10–10-2010	KB-61	Triangular dredge	337–343	B2	PX560216
Pvent_st2_24	Norway_2	Norway Korsfjorden	59º58’8.79’’ N	5º22’4.37’’ E	09–09-2016	ST2	Triangular dredge	97–332	B1	PX560212
Pvent_st2_25	Norway_3	Norway Korsfjorden	59º58’8.79’’ N	5º22’4.37’’ E	09–09-2016	ST2	Triangular dredge	97–332	B2	PX560202
Pvent_Sw_01	Sweden_1	Sweden	58°53’1.00’’ N	11°06’0.40’’ E	27–03-2019	NA	ROV	104	B2	PX560200
RB_01404	Rockall_1	Rockall Bank	57º25’1.55’’ N	13º27’6.35’’ W	02–05-2018	0818H027	Bottom trawl	180–181	B1	PX560210
RB_01405	Rockall_2	Rockall Bank	57º25’1.55’’ N	13º27’6.35’’ W	02–05-2018	0818H027	Bottom trawl	180–181	B1	PX560211
RB_01410	Rockall_3	Rockall Bank	57°23’6.40’’ N	13°55’3.80’’ W	03–05-2018	0818H027	Bottom trawl	145–147	B2	PX560203
RB_01809	Rockall_4	Rockall Bank	56º43’8.70’ N	14º44’2.10’’ W	25–09-2018	S18/344	Bottom trawl	188–195	B1	PX560204
RB_01811	Rockall_5	Rockall Bank	56º43’8.70’ N	14º44’2.10’’ W	25–09-2018	S18/344	Bottom trawl	188–195	B1	PX560205
RB_01819	Rockall_6	Rockall Bank	55º54’3.55’’ N	15º13’4.00’’ W	24–09-2018	S18/344	Bottom trawl	338–340	B1	PX560208
RB_01820	Rockall_7	Rockall Bank	55º54’3.55’’ N	15º13’4.00’’ W	24–09-2018	S18/344	Bottom trawl	338–340	B1	PX560209
Roscoff_1	Roscoff_1	Roscoff	48°43’54.26” N	04°3’32.67” W	05–05-2022	NA	SCUBA diving	30	B1	PX560198
Roscoff_2	Roscoff_2	Roscoff	48°43’54.26” N	04°3’32.67” W	05–05-2022	NA	SCUBA diving	30	B2	PX560199
Roscoff_3	Roscoff_3	Roscoff	48°43’54.26” N	04°3’32.67” W	05–05-2022	NA	SCUBA diving	30	B1	PX560217
SM_ROV26_16	Tromsø_1	Tromsøflaket	71º23’7.36’’ N	16º48’5.04’’ E	05–08-2017	ROV26	ROV	332	B2	PX560218
WTR_01266	WTR_1	Wyville Thomson Ridge	59º48’5.70’’ N	08º04’8.55’’ W	02–09-2018	WTR-S18/314	Bottom trawl	900	B1	PX560213

For morphological identification, we used a fragment of ~ 125 mm^3^ for spicule analyses. We incubated the sponge tissue overnight in sodium hypochlorite (NaClO) at room temperature to dissolve organic matter and release spicules. We sequentially washed the spicules with deionized water, 50% ethanol, and 96% ethanol. Then, we mounted the clean spicules on slides with DPX mounting medium for measurement using an Olympus BX43 compound microscope (Olympus Corporation, Japan) equipped with an Olympus UC50 camera and its associated software cellSens Standard v.1.16 (Olympus Corporation, Japan). All samples presented styles and sinuous strongyles as unique spicular types, which together with the sponges’ cup-shaped external morphology were consistent with their identification as *P. ventilabrum*.

### DNA extraction and sequencing

We used a small tissue fragment (~125 mm^3^) from each sample for DNA extraction using the DNeasy Blood & Tissue kit (QIAGEN, Valencia, USA), following the manufacturer’s instructions. DNA quality was assessed using a Nanodrop One spectrophotometer (Thermo Fisher Scientific), and concentrations were quantified using a Qubit 4 fluorometer with the Qubit dsDNA BR Assay kit (Invitrogen, Carlsbad, MCA, USA). DNA extractions were shipped to Novogene for Illumina whole-genome sequencing library preparation (350 bp insert size) and sequenced on a NovaSeq 6000 platform, targeting 30 Gb of 150 bp paired-end reads per sample.

### Sequence filtering, mitogenome assembly, and annotation

After sequencing, we processed raw sequencing data using trimmomatic v-0.39 [71] with default parameters to remove adapter sequences and low-quality reads. Filtered read quality was validated with fastqc v-0.12.1 [72]. We assembled the mitogenomes of all 28 specimens using novoplasty v-4.3.5 [73], providing filtered reads, the publicly available *P. ventilabrum cox1* partial sequence MT506485.1 as a seed, and setting a k-value of 33. As a reference, we selected the mitogenome assembly OY997403.1, since it was co-sequenced with the nuclear reference genome of *P. ventilabrum* using Oxford Nanopore Technology (ONT) long-read sequencing. Sequences used as references were not further included in subsequent analyses. To validate mitogenome assemblies, we mapped each specimen’s reads back to its respective mitogenome using bwa-mem [74] to verify uniform coverage across mitogenomes. We visualized coverage results using the package ggplot2 v-3.5.1 [75], implemented in r v-4.3.3. Finally, we annotated mitogenomes using mitos v-2.1.9 [76] through the Galaxy portal (https://usegalaxy.org/), and we manually curated them in geneious prime 2022.0.1 (Biomatters, Auckland, New Zealand) [77] to ensure the presence of start and stop codons on coding genes and the accuracy of rRNA and tRNA boundaries.

### Mitogenome diversity and intron validation

We aligned all 28 mitogenomes using mafft v-7.520 [78] under the L-INS-I algorithm, implemented in geneious prime. Two major mitochondrial structures were revealed in the alignment (see Results section), herein named “mitogroups”. Consequently, we carried out a cross-mapping using bwa-mem to validate the differential assembly. To do so, we mapped the reads from a representative specimen of mitogroup “A” (Deep_094_05) against the mitogenome of a mitogroup “B” specimen (NwK_KB_61_2) and vice versa. These two representatives were randomly selected from each mitogroup’s specimen pool. We used dnasp v-6 [79] to quantify the number of indels “AS IS” across the 28 mitogenomes alignment and in two regions of the mitogenome that structurally characterize each mitogroup (see Results section). Once evaluated, we excluded indels and calculated the number of polymorphic positions (S), number of haplotypes (h), nucleotide diversity (π), haplotype diversity (Hd), and Tajima’s D for the entire mitogenome alignment and each genic domain. We repeated the analyses at the mitogroup level. Finally, we calculated pairwise fixation indices (F_ST_) among mitogroups using whole mitogenomes and each genic domain, excluding gaps in comparisons.

For both mitogroups, the *16S* and *cox1* genes were annotated as two exons separated by a potential intron (see Results section). To evaluate whether the regions between exons corresponded to introns or were the result of an annotation artifact, we employed a complementary strategy based on 1) gene homology, 2) splicing corroboration, and 3) expression analyses based on intronic and exonic coverage. For the gene homology analysis, we retrieved the mitogenome of the haplosclerid *Amphimedon queenslandica* (DQ915601.1) [80], as its *cox1* gene is used as a reference for the detection and nomenclature of sponge introns. We then aligned the *cox1* and *16S* genes of *P. ventilabrum* against their orthologs in *A. queenslandica* using mafft v-7.520 and, for each reference gene, annotated the positions where introns were inserted. In parallel, we downloaded already published RNA-seq data (BioProject PRJNA576458) from a Norwegian *P. ventilabrum* specimen [41]. We applied read-quality filters with trimmomatic and mapped the resulting high-quality reads to mitogenome assemblies. To manually validate the splicing events in the *16S* and *cox1* genes, we used tablet v-1.21.02.08 to assess the quality of reads mapped to intron–exon boundaries and to determine whether reads were consistently split between the first and second exons. We visualized RNA coverage using the r package ggplot2 [75]. We conducted independent two-sample t-tests with the *t.test* function implemented in r to evaluate significant differences in nucleotide-level coverage between intronic and exonic regions for the *cox1* and *16S*. For *cox1*, we had 1036 nucleotides in exon1, 442 in exon2, and at least 2278 in the intron. For *16S*, we had at least 1404 nucleotides for exon1, 1032 for exon2, and at least 821 for the intron. Both the *cox1* and *16S* intronic sequences of *P. ventilabrum* were evaluated using RNAweasel (https://megasun.bch.umontreal.ca/apps/rnaweasel/) to classify them into group I or group II introns. Next, since most metazoan mitochondrial introns have a fungal origin [81], we used the mold protozoan mitochondrial and the alternative yeast nuclear genetic codes (translation tables 4 and 12, respectively), to look for the presence of open reading frames (ORFs) of at least 400 bp within both *16S* and *cox1* introns. To assess the origin of the intronic (group I or group II) and ORF regions, we used blast v-2.13.0 with the algorithm blastn [82] to identify homologous sequences belonging to other taxa in the core_nt NCBI database (accession date: 27/08/2025). We also carried out a blast search of the exonic regions for comparison. Finally, as many sponge mitogenomes in NCBI were classified as unverified and therefore excluded from the core_nt database, we downloaded all available Bubarida (same order), Biemnida (sibling order), and Axinellida (Heteroscleromorpha order in which mitochondrial introns have been reported) mitogenomes (accession date: 24/09/2025, Table S1) to build a local database with the blast makeblastdb option. We then compared our intronic sequences against this custom database using blast to assess the extent of intron distribution within these lineages.

### Haplotype networks, phylogeny, and lineage-specific mitochondrial dynamics

We constructed haplotype networks using popart v-1.7 [83]. We did so for (I) whole mitogenome assemblies to capture as much information as possible. Then, we carried it out for (II) the *cox1* first exon, as this exon encompasses the DNA barcode region, traditionally used for population genetics with Sanger sequencing. Finally, we constructed haplotype networks based on (III) the *nad2* gene, as this region showed the highest variability among mitochondrial genes (see Results section). We exported sequence alignments in Nexus format and assigned each sample to its respective locality. We generated our haplotype networks for all datasets using the TCS Network function implemented in popart [84].

We downloaded from NCBI the annotated mitogenomes of the bubarid species *Acanthella acuta* OM729606.2 [47], the suberitid species *Suberites domuncula* (Olivi, 1792) NC_010496.1 [85, 86], and the agelasid species *Cymbaxinella corrugata* (George & Wilson, 1919) NC_006894.1 [87, 88] and *Agelas schmidti* Wilson, 1902 NC_010213.1 [87, 89]. We selected these taxa to include a representative of a sister genus within the order Bubarida (*A. acuta*) and three outgroup taxa within Heteroscleromorpha belonging to orders sister to Bubarida (*S. domuncula*, *C. corrugata*, and *A. schmidti*), thereby enabling rooting of phylogenetic analyses. We independently aligned all orthologous protein-coding and ribosomal genes, except for the *cox1* and *16S*, to avoid biases produced by the presence of introns. Importantly, the *cox2* did not include *A. acuta*, as an intron has also been described in this gene for this species [47]. Moreover, we aligned whole mitogenomes with and without gappy regions (see Results section) to evaluate whether non-genic regions had a significant effect on recovering the phylogenetic relationships of *P. ventilabrum*. Notably, *S. domuncula* was excluded from the whole mitogenome analysis since it could not be aligned with the other mitogenomes due to severe structural rearrangements. We constructed ultra-fast Maximum Likelihood gene trees for each gene using iq-tree v-2.3.6 [90], using the whole gene sequence as a single partition. We predefined a GTR+I+G model for all genes, set 10,000 iterations of ultrafast bootstrap (ufBS), established *A. acuta* as the outgroup, and included the option “−bnni” to reduce the effect of ufBS on branch support. To avoid suboptimal topologies, we repeated each tree 10 times with the option “--runs”. A consensus supertree was inferred by compiling all gene trees using astral v-5.7.1 [91]. For the supermatrix approach, we concatenated all gene alignments to generate a phylogenetic tree with iq-tree, treating each gene as an independent partition and using the same parameters previously used for the gene trees. We visualized all resulting topologies with the itol v7 [92] web server (https://itol.embl.de/), and established ufBS above 90 as a threshold for inference of supported nodes.

To assess whether mitochondrial lineages differed in mitochondrial copy number (mitoDNA-CN), we mapped filtered reads from each specimen to the chromosome scaffolds of the *P. ventilabrum* v3 reference genome [93] and to their respective mitogenomes using bwa-mem. Mean coverage per sample for mitochondrial and nuclear mappings was obtained with the depth function in samtools. MitoDNA-CN was then estimated as the ratio of the mean mitochondrial to nuclear coverage, multiplied by two under the assumption of diploidy. Differences among groups were tested using Student’s t-test and the Kolmogorov–Smirnov test in r. MitoDNA-CN distributions were visualized as boxplots with ggplot2 in r.

## Results

### Insights from mitogenome assembly and annotation

**Fig. 2:**
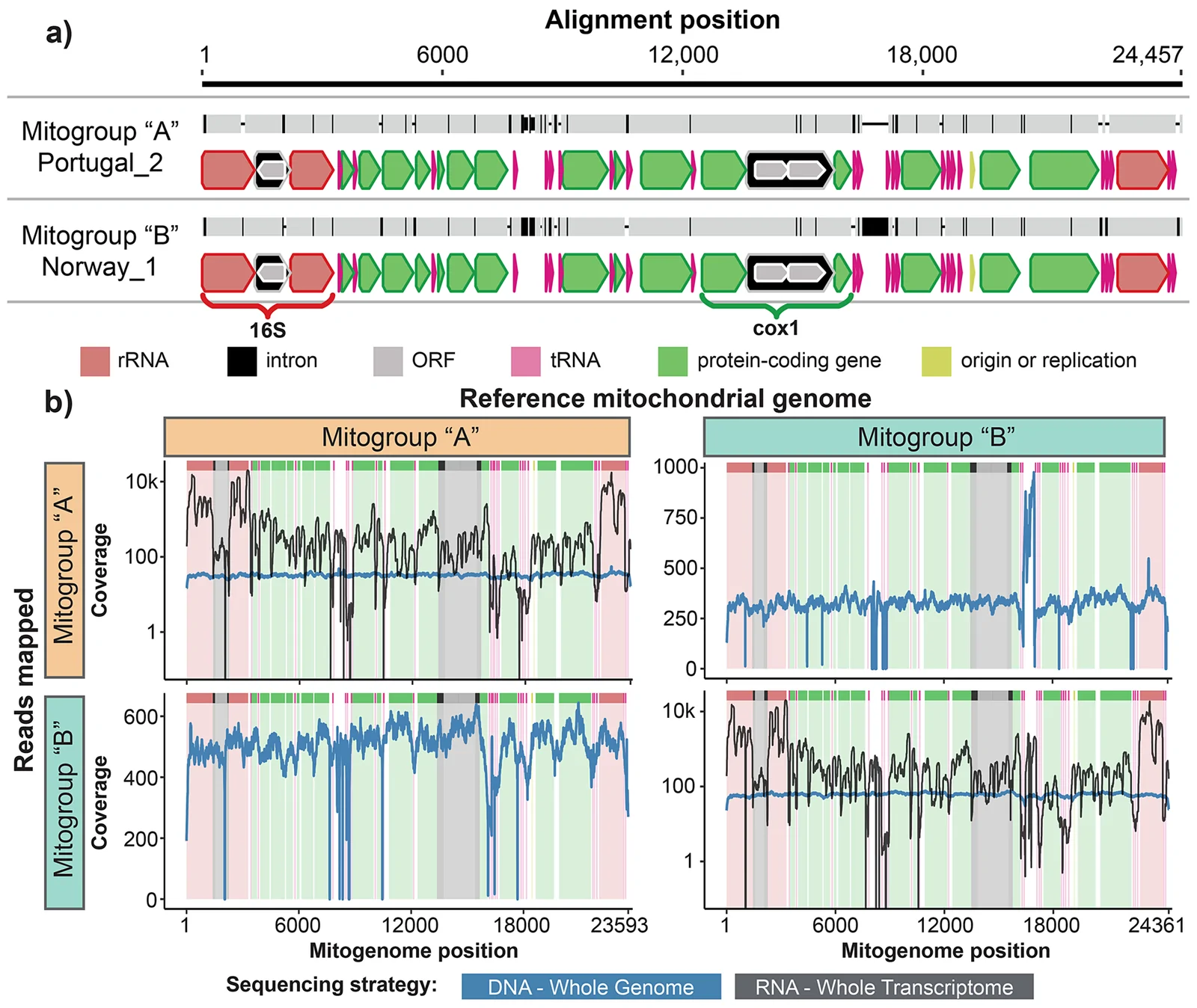
Mitogenome features. **a**) mitogroup “A” and “B” alignment. Grey boxes above the arrows indicate regions with 100% identity, black boxes indicate variants, and lines indicate gaps. The direction of the arrows indicate the direction of the gene. Genes hosting an intron (*16S* and *cox1*) are indicated. From left to right: *16S*-exon1, intron 16S_1647_[16S-ORF], *16S*-exon2, *tRNA-R, nad4L, tRNA-E, nad6, cox2, nad3, tRNA-K, atp8, atp6, cox3, tRNA-Q, tRNA-W, tRNA-N, tRNA-L1, cob, tRNA-T, atp9, tRNA-S1.1, nad4, tRNA-H, cox1*-exon1, intron cox_1141_[cox1-ORF_813_, cox1-ORF_876_], *cox1*-exon2, *tRNA-S2, tRNA-S1.2, tRNA-P, tRNA-A, tRNA-C, nad1, tRNA-D, tRNA-L2, tRNA-I, tRNA-M.1, nad2, nad5, tRNA-M.2, tRNA-Y, tRNA-F, 12S, tRNA-G, tRNA-V*. **b**) mitogroup “A” and “B” back-mapping and cross-mappings of DNA (blue line) and RNA (black line) reads. For accurate visualization of RNA expression levels, the “y” axis of the back-mapping panels are log10-scaled, whereas cross-mapping panels are not since they only show WGS coverage. Note that the specimens with short IDs Portugal_2 and Norway_1 were selected at random as representative of their respective groups

We received, on average, 6.6 Gb of raw reads per sample, which were reduced to ~6.3 Gb after quality filtering (Table 2). All mitogenomes from all specimens were successfully circularized, displaying high coverage uniformly across assemblies. We identified two structurally divergent mitochondrial haplogroups (hereafter called mitogroups) (Fig. 2a). One of them, herein cataloged as mitogroup “A”, had shorter assemblies (23,559– 23,593 bp) with a GC content of 39.4% (Table 2) and was found exclusively in Portugal and the Cantabrian Sea (southernmost distribution of the samples). The other structure, herein cataloged as mitogroup “B”, presented larger assemblies (24,308– 24,371 bp) with a GC content of 39.3%–39.4% (Table 2) and was spread across all northern localities (Fig. 2a). Backmapping of the reads confirmed uniform high coverage across assemblies (Fig. 2b). On the other hand, cross-mappings showed consistent coverage across assemblies, except for small regions with zero coverage, indicating no mapping of reads from the homologous mitochondrial structure (Fig. 2b). When mapping the mitogroup “A” reads against the mitogroup “B” assembly, a region situated in positions 16,531– 17,214 showed coverage three times greater than the baseline, suggesting that the mitogroup “A” assembly circularized without resolving a large region homologous to the mitogenome “B” that is likely present in three copies (Fig. 2b). To capture longer repetitive regions, we attempted to reassemble mitogroup “A” specimens using k-mers of 150, 145, 140, 135, 130, and 125 bp. These attempts remained unsuccesful, confirming that with the current data no better mitogenome “A” can be assembled and, therefore, the possibly three-times copied region of mitogroup “A” (compared to mitogroup “B”) cannot be resolved.

**Table 2:** Sample sequencing features for the 28 specimens included in the study. We provide their sampling campaign ID, short ID, ENA BioSample code, number of raw and filtered sequenced base pairs, mitochondrial assembly size, GC content, and group nomenclature, and length of the size-variable *16S* large exon, *16S* intron and cox1 intron

Sample ID	Short ID	BioSample	Raw reads (in bp)	Filtered reads (in bp)	Nuclear coverage	Assembly size	GC content	Mitochondrial coverage	Mitochondrial copy number	Mitochondrial group	Mitogenome accession number	16S large exon lenght	16S intron length	cox1 intron lenght
CS_DR1_2_1	Cantabria_1	SAMEA120390426	6806342700	6542801698	15.7	23,592	39.4	357.5	45.4	mitoA	PX560193	1404	821	2286
CS_DR1_2_3	Cantabria_2	SAMEA120390427	5326443600	5118594930	15.4	23,593	39.4	279.5	36.2	mitoA	PX560194	1404	852	2286
CS_DR8_60	Cantabria_3	SAMEA120390428	7436648700	7124740993	8.0	23,559	39.4	153.6	38.6	mitoA	PX560191	1404	852	2286
Deep_094_02	Portugal_1	SAMEA120390429	6270880500	6020753158	17.8	23,593	39.4	284.0	31.9	mitoA	PX560195	1404	852	2286
Deep_094_05	Portugal_2	SAMEA120390430	6392860200	6117674088	18.3	23,593	39.4	327.4	35.7	mitoA	PX560196	1404	852	2286
Deep_094_06	Portugal_3	SAMEA120390431	6083238900	5875067558	19.2	23,570	39.4	280.2	29.2	mitoA	PX560192	1404	852	2286
Dstrait_3	Strait_3	SAMEA120390452	4074995100	3867485245	13.9	24,344	39.3	243.3	35.0	mitoB1	PX560206	1405	830	2286
ME85_1082_D	Iceland_1	SAMEA120390432	9346127100	9050081317	25.5	24,361	39.3	302.3	23.7	mitoB1	PX560214	1405	830	2286
ME85_1082_E	Iceland_2	SAMEA120390433	8418283500	8128290855	23.2	24,338	39.3	308.1	26.5	mitoB1	PX560201	1404	830	2286
ME85_1082_F	Iceland_3	SAMEA120390434	7862205900	7594482897	20.2	24,361	39.3	297.0	29.5	mitoB1	PX560215	1405	830	2286
NwK_KB_61_2	Norway_1	SAMEA120390435	8261831400	7985575735	27.8	24,361	39.3	505.8	36.4	mitoB1	PX560216	1405	830	2286
Pvent_st2_25	Norway_3	SAMEA120390437	10748000000	10432752437	35.3	24,344	39.3	944.5	53.5	mitoB1	PX560202	1405	830	2286
Pvent_Sw_01	Sweden_1	SAMEA120390438	7329102300	7048804549	20.3	24,329	39.3	742.2	73.2	mitoB1	PX560200	1405	830	2286
RB_01410	Rockall_3	SAMEA120390441	8129946900	7811116862	26.1	24,344	39.3	457.3	35.0	mitoB1	PX560203	1405	830	2286
Roscoff_3	Roscoff_3	SAMEA120390448	6083653800	5830073325	9.0	24,313	39.3	376.6	84.0	mitoB1	PX560217	1444	821	2287
SM_ROV26_16	Tromsø_1	SAMEA120390449	8181904200	7935554970	23.6	24,371	39.3	829.5	70.2	mitoB1	PX560218	1405	830	2286
Roscoff_1	Roscoff_1	SAMEA120390446	5756695500	5304768115	20.1	24,313	39.3	533.6	53.2	mitoB2	PX560198	1444	821	2287
Dstrait_1	Strait_1	SAMEA120390450	4487879100	4258172878	13.9	24,308	39.3	436.0	62.7	mitoB2	PX560197	1444	821	2278
Dstrait_2	Strait_2	SAMEA120390451	4714671000	4473200736	17.2	24,348	39.4	343.7	40.0	mitoB2	PX560207	1444	821	2287
Pvent_st2_24	Norway_2	SAMEA120390436	7468183500	6964061225	25.7	24,360	39.4	663.6	51.6	mitoB2	PX560212	1443	821	2287
RB_01404	Rockall_1	SAMEA120390439	10259359500	9983885132	26.7	24,359	39.3	319.7	23.9	mitoB2	PX560210	1444	821	2287
RB_01405	Rockall_2	SAMEA120390440	11133868500	10867893993	32.1	24,359	39.3	346.9	21.6	mitoB2	PX560211	1443	821	2287
RB_01809	Rockall_4	SAMEA120390442	3493052700	3283458528	13.9	24,344	39.3	285.6	41.1	mitoB2	PX560204	1444	821	2287
RB_01811	Rockall_5	SAMEA120390443	3389718000	3202863273	13.2	24,344	39.3	287.4	43.5	mitoB2	PX560205	1444	821	2287
RB_01819	Rockall_6	SAMEA120390444	3083450700	2913988475	11.9	24,349	39.4	266.3	44.9	mitoB2	PX560208	1444	821	2287
RB_01820	Rockall_7	SAMEA120390445	3970390800	3766700481	14.2	24,349	39.4	467.4	65.8	mitoB2	PX560209	1444	821	2287
Roscoff_2	Roscoff_2	SAMEA120390447	5225206800	4974873833	22.9	24,371	39.3	652.1	56.9	mitoB2	PX560199	1405	830	2286
WTR_01266	WTR_1	SAMEA120390453	4033882200	3835863533	10.7	24,360	39.4	610.6	114.7	mitoB2	PX560213	1444	821	2287

Mitochondrial annotations revealed the presence of 14 protein-coding genes (*atp6, atp8, atp9, nad1, nad2, nad3, nad4, nad4l, nad5, nad6, cox1, cox2, cox3*, and *cob*), 2 ribosomal genes (*16S* and *12S*), and 24 tRNA genes, typically found in most sponges (Fig. 2a). The *cox1* gene was architecturally composed of a large exon of 1,141 bp, followed by a large intron of 2,275–2,284 bp (Table 2), and ending with a small exon of 420 bp. Similarly, the *16S* gene comprised a first large exon ranging from 1,404 to 1,444 bp (depending on the mitogenome according to the presence of indels, Table 2), an intron of 821 or 830 bp for mitogroup “B” and 834 or 852 bp for mitogroup “A” (Table 2), and a second exon of 1,032 bp (Fig. 2a).

### Structural and nucleotide mitochondrial diversity

The two mitogroups were structurally differentiated by the presence of 1269 indels. Yet, they primarily differed by a region of 1032 bp (alignment positions 7,923–8,955), which encompassed 57 non-overlapping short indels spanning 238 bp, and a large indel of 683 bp (mitogenome alignment positions 16,531– 17,214) (Fig. 2a, Figure S1). The 683 indel was located in the intergenic region between tRNA-S1 and tRNA-P, and the 57 short indels were located in the intergenic regions of tRNA-Q, tRNA-W, tRNA-R, and tRNA-L1. None of their locations compromised tRNA structure, gene order, regulatory regions, or coding potential. Both the 683 bp large indel and the 57 short indels were caused by the insertion of simple random sequences, as we found no repetitive motifs present elsewhere within the mitogenomes. Notably, in both indel-rich regions, indels were polymorphic across samples within each group (Figure S1). Across whole mitogenomes, both mitogroups displayed within-group structural mutations, with 94 indels in mitogroup “A” and 461 indels in mitogroup “B”. Mitogroup “B” could be further subcatalogued into two groups, herein named as mitogroups “B1” and “B2”, also driven by differences in these two indel-rich regions.

The alignment of all 28 mitogenomes revealed 120 segregating sites (S), defining 23 haplotypes (h) (Table 3). Hd was high (0.987), whereas π remained low (0.00178). Tajima’s D was strongly positive (13.452) (Table 3). Protein-coding genes showed generally low S and π values, with all mutations being synonymous. Among them, *nad2* exhibited the highest variability (S = 4; π = 0.002) (Table 3). The *16S* rRNA gene contained more segregating sites than protein-coding genes (16S-exon1 = 7 + 16S-exon2 = 4), while intronic regions displayed the greatest variation overall, with 7 segregating sites in the *16S* intron and 10 in the *cox1* intron (Table 3). The *nad2* also exhibited the highest Tajima’s D among genic regions (24.744). When analyzed by mitogroup, S decreased markedly compared to the full dataset (Table S2), accompanied by reductions in Hd and π across all groups. Tajima’s D values also declined, becoming strongly negative in most cases, except for the “B2” mitogroup in the 16S-exon1 (Table S2). Pairwise F_ST_ estimates based on whole mitogenomes indicated substantial genetic differentiation among mitogroups, with all pairwise comparisons exceeding 0.5 (Table S3).

**Table 3:** Diversity statistics and RNA expression. For each region being analyzed, we provide the number of polymorphic positions (S), number of haplotypes (h), nucleotide diversity (π), haplotype diversity (Hd), Tajima's D, and the mean RNA coverage based on specimen Norway_61_2 as a proxy for expression. Note that genes hosting an intron have been separated as -exon1, -intron, and -exon2

Region	S	h	π	Hd	Tajima’s D	mean RNA coverage
mitogenome	120	23	0.00178	0.987	13.452	10527.9
12S	1	2	0.00011	0.138	−0.741	51709.5
16S-exon1	8	7	0.00260	0.833	23.837	35956.3
16S1647	7	7	0.00214	0.780	−0.128	1304.0
16S-exon2	3	4	0.00087	0.690	0.392	61927.8
atp6	1	2	0.00055	0.349	0.572	2359.1
atp8	0	1	0.00000	0.000	n.d.	4141.0
atp9	0	1	0.00000	0.000	n.d.	5566.8
cob	1	2	0.00042	0.476	13.604	5939.9
cox1-exon1	0	1	0.00000	0.000	n.d.	6944.4
cox1141	10	10	0.00138	0.886	0.727	2066.1
cox1-exon2	1	2	0.00120	0.508	15.574	11071.0
cox2	1	2	0.00063	0.476	13.604	4507.8
cox3	1	2	0.00064	0.508	15.574	4782.4
nad1	2	3	0.00052	0.455	−0.111	689.9
nad2	4	3	0.00204	0.667	24.744	2124.7
nad3	0	1	0.00000	0.000	n.d.	2246.5
nad4	1	2	0.00028	0.349	0.572	1828.1
nad4l	1	2	0.00169	0.508	15.574	7379.1
nad5	3	4	0.00040	0.563	−0.267	2942.4
nad6	2	3	0.00114	0.548	0.266	10849.6

### RNA-seq and orthologs to validate intronic regions

The *cox1* intron was found to be inserted, by comparison, at position 1,141 of the reference *A. queenslandica cox1* gene (hereafter cox_1141_, following previously established sponge intron nomenclature [47, 53]), and was assigned by RNAweasel to intron group II. Its 5’ motif was “GTGCG”, and its 3’ motif was “AC”, coinciding with the 5’ and 3’ motifs of group II introns “GUGYG” and “AY”, respectively [94]. Using the alternative yeast nuclear genetic code, two ORFs of 813 and 876 bp, respectively (from now on cox1-ORF_813_ and cox1-ORF_876_), were identified within the *cox1* intron. The *16S* intron was found to be inserted, by comparison, at position 1,647 of the reference *A. queenslandica 16S* gene (hereafter 16S_1647_), and RNAweasel could not assign it to either group I or group II intron. However, its 5’ and 3’ motifs were “GTGCT” and “AT”, as described for group II introns. Using the mold protozoan mitochondrial genetic code, we identified a large ORF of 648 bp in the reverse strand of the *16S* intron (hereafter 16S-ORF). The mapping of RNA-seq data onto our mitochondrial groups corroborated, in both mitogroups, a variable coverage across the assembly, indicating differential expression levels by region (Fig. 2b, Table 3). Overall, ribosomal regions had the highest coverage (48,476X), followed by protein-coding regions (4,264X), and tRNA (184X) (Fig. 2b). The intronic region of the *16S* showed significantly lower coverage (1,304X) than the exons (~46,730X) (*t* = −33.053, df = 1237.6, *p*-value < 2.2e^−16^), similarly to what we observed for *cox1* (intron = 2,066X; exons = 8,140X; *t* = −29.852, df = 766.3, *p*-value < 2.2e^−16^) (Table 3). Finally, both intron boundaries could be further corroborated by manually checking the read mapping across the exon1-exon2 boundaries, since many reads were consistently split between the first and second exon (Figure S2).

For the *16S*, the 100 best BLAST hits of the first exon region returned other poriferan species, with query covers of 29–100% and percentages of identity ranging from 72.8–100% (Table S4). For the second exon, 96 hits of the top 100 returned poriferan species (cover = 63–100%; ID = 80.2–99.9%), whereas 4 returned cnidarian species (cover = 78–81%; ID = 76.7–77.3%) (Table S5). The intronic region of the *16S* (16S_1647_) returned a total of 57 hits. From these, only five hits corresponded to poriferan species (Table S6) belonging to two *Phakellia ventilabrum, Acanthella acuta*, *Phakellia robusta* Bowerbank, 1866 [95], and *Cymbastela* sp. (identified as *Cymbastela concentrica* in NCBI, probably misidentified (Merrick Ekins, pers. comm.)). All of these species belong to the order Bubarida [96] (Table S6). These taxa displayed coverage values ranging from 76% to 100%, and percentages of identity from 66.4% to 96.2% (Table S6). The remaining hits returned three placozoan species (cover = 9%; ID = 77.22%), and 64 *Saccharomyces* species (cover = 4%; ID = 94.44%). The 16S-ORF only returned the same sponge hits as the intron, although the percentage of coverage shifted to 69–100%, and the percentage of identity was 66.1–95.1% (Table S7). The 16S_1647_ was not found in any other mitogenome of the local database. For the *cox1*, the BLAST carried out for the first exon region returned exclusively other poriferan species among the 100 best hits, with query covers of 58–100% and percentages of identity ranging from 81.6–100% (Table S8). For the second exon, all hits were against poriferans with covers of 94–100% and percentages of identity of 78.3–100% (Table S9). The group II intronic region cox_1141_ only returned four hits against poriferan species (Table S10). We only detected a homolog cox_1141_ structure for *P. ventilabrum* (cover = 100%; ID = 99.72–99.74%), *A. acuta* (cover = 100%; ID = 94.1%), and *Cymbaxinella verrucosa* (Esper, 1794) [97] (cover = 98%; ID = 67.1%); these species belong to the orders Bubarida and Agelasida [96]. Eighty-five out of the 96 remaining hits returned species of Fungi (Ascomycota and Basidiomycota), with query covers of 2–7% and 87.1–100% identity (Table S10). The remaining hits corresponded to algae and bacterial species with 73.4–81.6% identity. The cox1-ORF_813_ returned 72 hits (Table S11), most of them corresponding to bacterial species (cover = 6–23%, ID = 67.7–87.5%), and only four hits corresponded to sponge taxa (cover = 100%, ID = 69.6–100%). The cox1-ORF_876_ returned 5 hits in total (Table S12), four of them corresponding to poriferan species (cover = 75–100%, ID = 64.1–99.8%), and a single Ochrophyta hit (cover = 11%, ID = 73.5%). The cox_1141_ was found, based on our mitogenome of the local database, in the scopalinid species *Svenzea flava* (Lehnert & van Soest, 1999), with a coverage of 55% and an ID of 83.3%.

### Haplotype network and mtDNA copy number support differential lineages

**Fig. 3:**
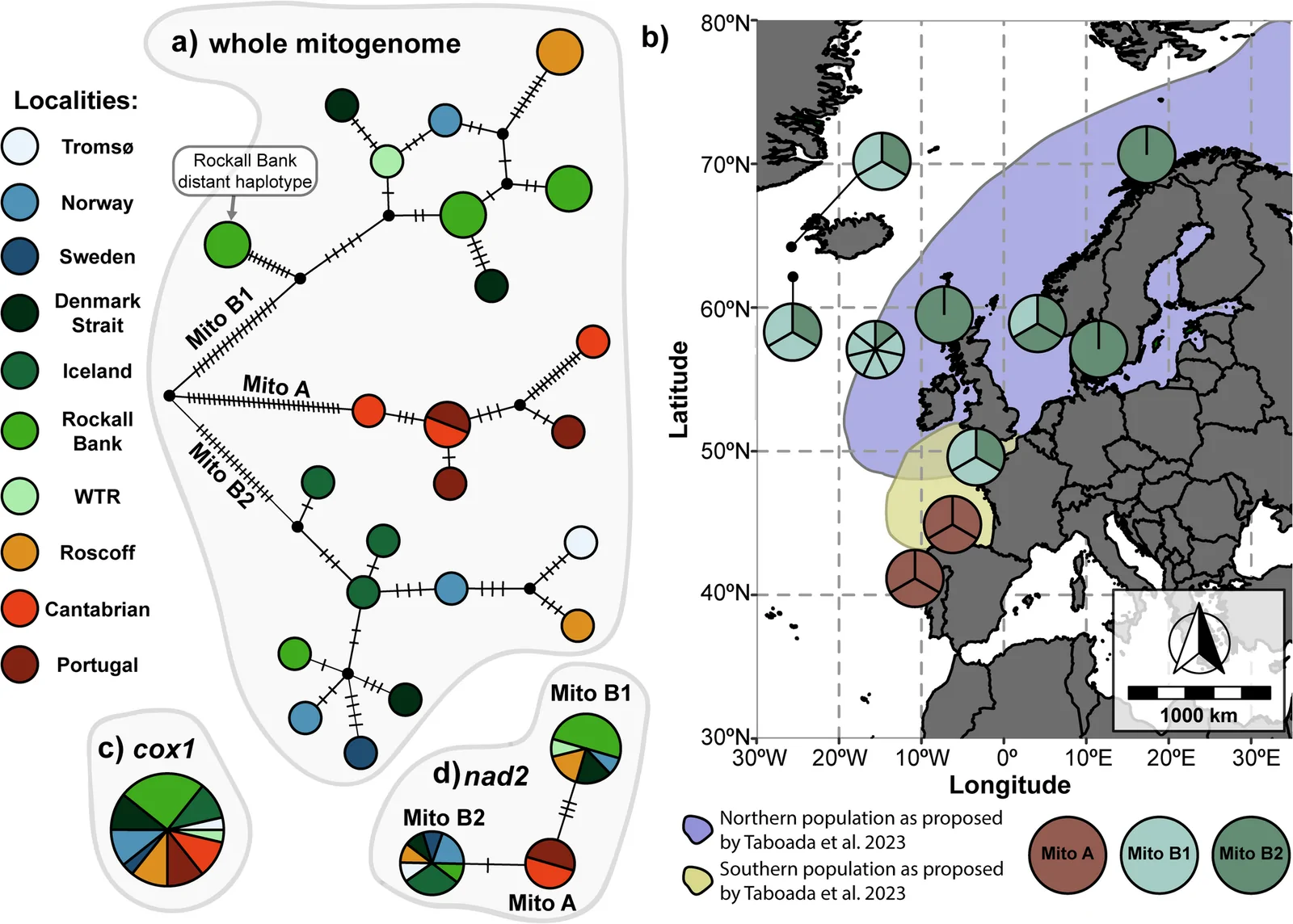
Genetic diversity of *Phakellia ventilabrum* based on mitochondrial data. **a**) haplotype network using whole mitogenome sequences. **b**) geographic representation of mitogroups “A”, “B1” and “B2” across their distribution range. Note that the colored areas correspond to the population structure proposed by Taboada et al. 2023 based on ddRadseq data. Note that circle size does not correspond to the number of specimens. **c**) haplotype network using the *cox1* gene first exon. **d**) haplotype network using the *nad2* gene. Circles in the haplotype networks in figures a-c are proportional to the number of specimens; hatch marks on branches correspond to the number of mutational steps between haplotypes, and missing inferred haplotypes are represented as small black circles

The haplotype network using whole mitogenomes (and therefore considering both nucleotide and structural mutations, treated pairwise) returned three main groups, with most haplotypes present in only one specimen, and only a few haplotypes shared by two specimens (Fig. 3a,b). One group was composed exclusively of specimens of mitogroup “A”, corresponding to the southernmost localities of Portugal and the Cantabrian Sea (Fig. 3a,b). One of the five haplotypes present in these southernmost localities was shared between these two localities, being the only non-private allele of the study (Fig. 3a,b). Separated from these southern localities by at least 48 mutations, we found a group composed of specimens belonging to the mitogroup “B1”, encompassing a haplotype carried by two specimens from Roscoff, three haplotypes shared by pairs of specimens from Rockall Bank, one haplotype from a specimen from WTR, two haplotypes from two Denmark Strait specimens, and finally one haplotype from one specimen from Norway (Fig. 3a,b). Within mitogroup “B1”, one haplotype from Rockall Bank was highly divergent from the other two haplotypes from Rockall Bank in the group, separated by at least 17 mutations. Finally, separated from the mitogroup “A” by at least 50 mutations, we found all specimens carrying the mitogroup “B2”, comprising the haplotype of a single sample from Roscoff, another from Rockall Bank, and then the haplotypes of the majority of the northernmost specimens (Fig. 3a,b). Among the latter, we recovered two samples from the Denmark Strait, one from Sweden, three from Norway (Western and Northern regions), and three from Iceland. Mitogroup “B1” and mitogroup “B2” were separated from each other by at least 37 mutations (Fig. 3a,b).

The *cox1* gene, with 1,108 bp, recovered a single haplotype (Fig. 3c). Conversely, the *nad2* gene, encompassing 997 bp, was the only molecular marker capable of distinguishing the mitogroups “A”, “B1”, and “B2”, although we recovered only a single haplotype for each mitogroup (Fig. 3d). With the *nad2* gene, mitogroup “B1” was separated from mitogroup “A” by three mutations and from mitogroup “B2” by four mutations (Fig. 3d). Mitogroups “A” and “B2” were separated by a single mutation.

The mitoDNA-CN of samples ranged from 21.6 to 114.7 (average = 46.6; standard deviation = 20.9) (Table 2, Figure S3). Across all comparisons, we found significant differences in mitoDNA-CN between mitogroups “A” and “B” based on the t-test, and between mitogroups “A” and “B2” with the KS-test (Figure S3, Table S13).

### Phylogenetic framework of *Phakellia ventilabrum*

**Fig. 4:**
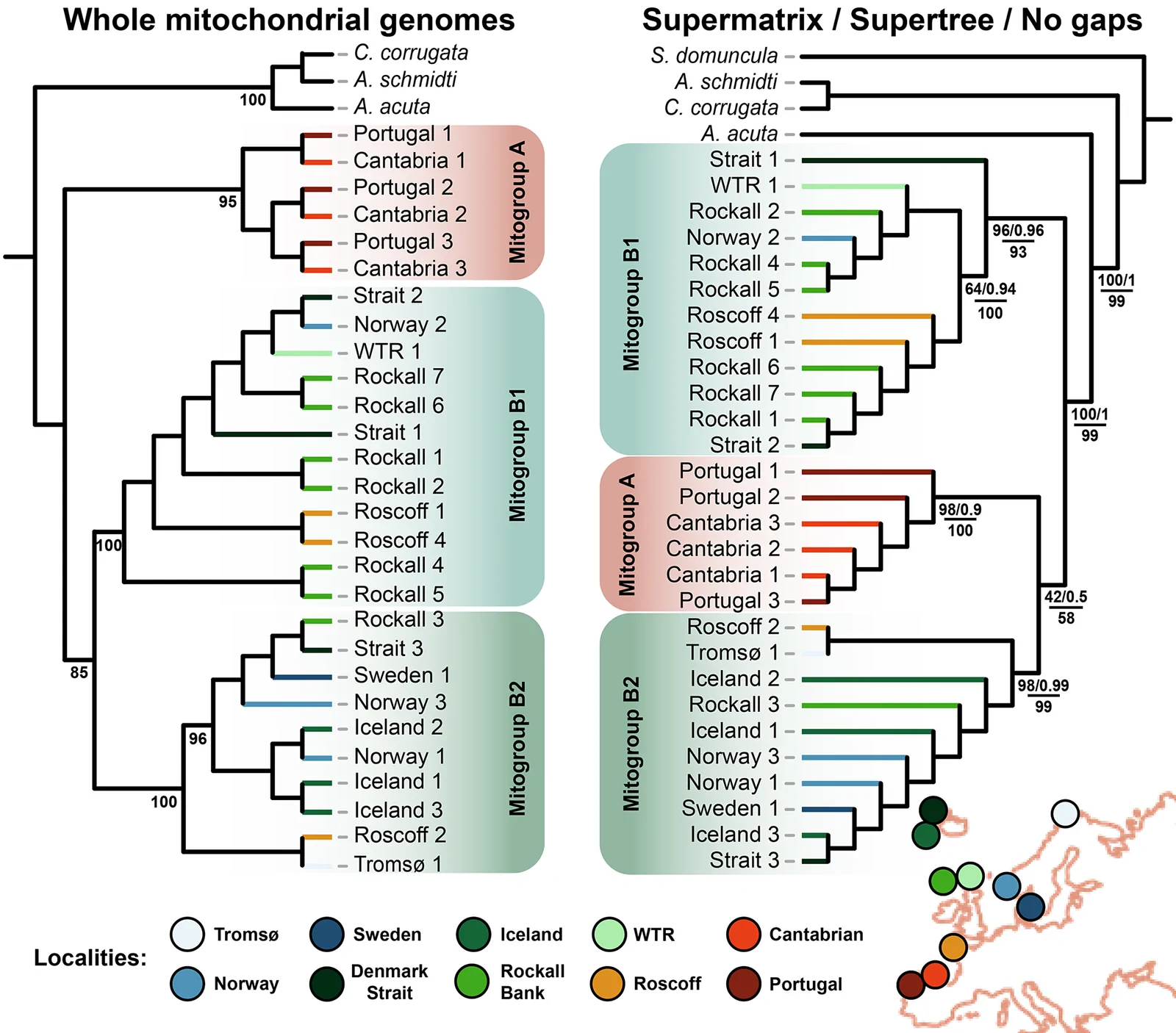
Phylogenetic relationships among the 28 *P. ventilabrum* sequences. Note that BS values are only represented for major lineages. The whole mitogenome tree lacks *S. domuncula* due to major rearrangements that prevented correct alignments. On the Supermatrix / supertree / No gaps topology, values above the horizontal lines near nodes indicate their support for the supermatrix and supertree using coding genes, but the *cox1* and the 12S. Values below the horizontal lines near nodes indicate their support for the whole mitogenome without gappy regions

We recovered two different topologies, depending on the phylogenetic approach carried out. When evaluating whole mitogenomes, the mitogroup “A” lineage was sister to mitogroups “B1” and “B2”, with mitogroups “B1” and “B2” as sister lineages supported by moderate node support (BS = 85). On the other hand, the phylogenetic reconstruction using the supermatrix (constructed by concatenating 13 protein-coding genes and one rRNA, resulting in 11,058 bp), supertree (based on the consensus topology derived from each of the 13 protein-coding genes and one rRNA), and whole mitogenome sequences after excluding gappy regions and rearranged regions revealed three monophyletic lineages that corresponded to mitogroup “A” (Supermatrix BS = 98, ASTRAL support = 0.9, no-gaps BS = 100), mitogroup “B1” (Supermatrix BS = 96, ASTRAL support = 0.96, no-gaps BS = 93), and mitogroup “B2” (Supermatrix BS = 98, ASTRAL support = 0.99, no-gaps BS = 99) (Fig. 4). Mitogroup “A” and mitogroup “B2” consistently appeared as sister lineages, although with no node support (Supermatrix BS = 42, ASTRAL support = 0.5, no-gaps BS = 58) (Fig. 4). These two lineages were sister to the mitogroup “B1”, with high node support (Supermatrix BS = 100, ASTRAL support = 1, no-gaps BS = 99). Within each mitogroup, most relationships among specimens were not supported, possibly due to lineage-specific mitochondrial homogeneity (Figure S4).

## Discussion

The rise of whole-genome sequencing techniques has transformed biological research, allowing a deeper understanding of keystone species’ evolution, adaptation mechanisms, population dynamics, and connectivity patterns—critical insights for the effective development of conservation management strategies [98]. Our study, by revisiting 28 specimens previously analyzed using RADseq to complement them with intraspecific mitogenomic information, has been key to shedding light on the genetic differentiation of *Phakellia ventilabrum* across the North Atlantic Ocean (Figs. 3, 4). This approach successfully overcame the limitations of traditional single-marker methods, such as *cox1*, which in general exhibits low intraspecific variability in demosponges [22, 57, 61] and, in particular, no variability in *P. ventilabrum*. When using whole mitogenome analysis, we revealed genomic differentiation between southern and northern sampling sites, consistent with a previous study using ddRADseq [19]. We identified two main distinct mitochondrial architectures (mitogroup “A” and mitogroup “B”) in *P. ventilabrum*, which are primarily differentiated by a large indel of ~ 680 bp (Fig. 2). The two mitochondrial structures share the same gene order, which is not conserved across demosponges [41]. Interestingly, mitogroup “A” exhibited significantly lower mitoDNA-CN than mitogroup “B”, indicating that the two mitogroups differ not only in mitochondrial genome structure but also in copy number. For the northern sampling sites, we found two sympatric lineages: mitogroup “B1” and mitogroup “B2”. Moreover, RNA-seq data supports the splicing functionality of the two mitochondrial introns reported in this study (the second case for *16S* in metazoan species to date).

### Intron acquisition and splicing functionality

Mitochondria are widely regarded as remnants of ancestral bacteria that established a highly integrated host-symbiont relationship [99]. Since introns are absent in ~99% of prokaryotic species, mitochondria have been historically assumed to lack them [100]. Nevertheless, introns play a crucial role in eukaryotes by allowing the emergence of alternative engines for evolution, permitting alternative splicing of genes and new regulatory pathways [101]. Sponges, along with placozoans and some cnidarians, represent an exceptional case among metazoans, as introns have been identified in several mitochondrial genes, such as *cox1*, *cox2*, and *16S* [48, 50, 56, 102]. These intronic regions could be identified given the absence of orthologous regions in closely related species, the presence of in-frame stop codons, exon-exon boundaries, differential coverage, and by the fact that they often return BLAST hits from diverse unrelated taxa, typically Fungi [48, 50, 56] but also placozoans (Kelly & Cárdenas, 2016). Two intron acquisition mechanisms have been reported for *cox1* in sponges: group I and group II introns, which differ in their secondary structures and self-splicing mechanisms [48]. The resemblance of sponge *cox1* introns to fungal sequences has led to the hypothesis that sponge introns originated via horizontal gene transfer with ancestral symbiotic fungal species [46, 54]. Our cox_1141_ returned BLAST hits against *A. acuta*, *C. verrucosa,* and *S. flava,* with query cover values above 75%, as well as many yeast species, although with query covers below 25%. Future research with a wider taxon sampling should aim to evaluate how abundant the cox_1141_ is across Porifera and provide more robust insights into its potential donor symbiont.

In contrast with protein-coding genes, where the presence of stop codons within a gene suggests the presence of putative introns, ribosomal introns are more challenging to identify. Since translation does not occur on ribosomal genes and therefore stop codons are commonly found within them, the presence of additional nucleotides with (or without) stop codons is often assumed to be insertions of loops within the secondary structure of the ribosome. Even so, this study stands out by using RNA-seq data to overcome this limitation, as we have successfully validated through exon-exon boundary assessment and differential expression the presence of the intron 16S_1647_. The 16S_1647_ BLAST displayed four hits on bubarid species (genera *Phakellia*, *Acanthella*, *Cymbastela*). Although RNAweasel failed to classify this intron as group I or group II, we found the typical splicing motif of group II introns flanking the 16S_1647_, and the presence of an ORF within it that might enable its spread across taxa. The BLAST hits of the 16S_1647_ against three placozoan species and many mitogenomes of *Saccharomyces* support this hypothesis. Moreover, the presence of a group II intron in the *16S* gene (yet with boundaries different from those found in the present study) was already suggested in *A. acuta*, although no ORF was found within it [47]. In contrast, we could find the 16S_1647_ ORF of *P. ventilabrum* using the alternative mold mitochondrial genetic code. Interestingly, this genetic code differs from that used to detect both cox_1141_ introns, suggesting two different donor symbionts. To date, only the *16S* intron of *A. acuta* has been described. Interestingly, besides the *P. ventilabrum 16S* intron, we also identified an additional BLAST hit in *Cymbastella* sp. Its mitogenome was recently assembled alongside its chromosome-level genome (https://portal.aquaticsymbiosisgenomics.org/data/root/details/Cymbastela%20concentrica), but remains unannotated. Consequently, our study increases the number of identified *16S* introns in sponges from one to three. However, as this manuscript focuses on *P. ventilabrum*, future work is needed to formally describe and characterize the putative 16S intron in *Cymbastella* sp.

Although several introns have been reported in sponges, as well as the putative organism transferring them, their splicing functionality remained untested. RNA-seq has been demonstrated to be a useful approach for addressing this issue. However, to apply this methodology, many cautionary measures must be taken into account, as factors such as RNA degradation during sample collection or preservation, biases introduced during RNA extraction or sequencing, and transcriptional noise can obscure and compromise the accurate interpretation of transcriptional profiles. For this study, the RNA from the *P. ventilabrum* specimen used for RNA-seq was snap-frozen immediately after collection, and gene expression profiles were consistent with those observed in other demosponge species as reported by Plese et al., (2021), suggesting that RNA degradation and sequencing biases were minimal. To map RNA-seq data on our *P. ventilabrum* mitogenomes provided strong evidence, based on coverage shifts (Fig. 2) and exon-exon boundaries, for correct intron annotation beyond the GTGCG-AC splicing motifs of group II introns [94], and confirmed that exons consistently exhibit significantly higher transcription than introns. Intronic regions in *P. ventilabrum* did not exhibit zero transcriptome coverage, but averaged ~1,300X (Fig. 2). Group II introns are active mobile genetic elements that self-splice after transcription by creating six structural domains (DI–DVI) that form a catalytic core remarkably similar to the spliceosome [94]. The coverage pattern and exon-exon boundaries, coupled with the presence of ORFs in both introns, may reflect that the group II intron is functional and capable of autosplicing. However, we cannot rule out the possibility that these ORFs are inactive, since the sponge mitochondrial genetic code differs from that of alternative yeast or molds that use both *cox1* and *16S* ORFs (necessary for intron self-splicing), and therefore their translation might not occur successfully.

In the scenario of inactive ORFs, the observed coverage pattern may reflect the normal transcription–translation–degradation cycle of multiexonic genes, produced by sponge-origin spliceosomes. In eukaryotic multiexonic genes, transcription occurs across entire genic regions, and once fully transcribed, the RNA is subsequently matured through splicing and polyA tailing [103]. After maturing, spliced intronic chains are degraded by exosomes and other proteins, while mature mRNA will be translated once or multiple times before being degraded [104]. Consistent with this model, the *cox1* exons had a coverage fourfold that of the intron cox_1141_ (Fig. 2), indicating that exon transcripts persist longer over time for translation, whereas intronic sequences rapidly degrade. Similarly, the *16S* exonic coverage is almost forty times higher than the 16S_1647_ coverage (Fig. 2). This extreme feature is possibly explained by the fact that rRNA genes’ functionality depends directly on the secondary structure of the RNA chains, which is not degraded after transcription [105]. As a result, *16S* exonic transcripts are kept without translation, whereas the intronic region is removed and degraded. We encourage future studies combining RNA-seq data and multiple mitogenomes hosting introns in all different evolutionary stages after their insertion (active ORF fused with the host gene exons, active ORF separated from the host gene exons, inactive conserved ORF, inactive degenerated ORF) [106], to understand better how group I and group II introns behave in hosts with different genetic codes than the donor across different degrading stages.

### Mitogenome structural vs. SNP evolution

Recent advances in mitochondrial evolution indicate that intraspecific diversity greatly exceeds previous knowledge, as more and more species with multiple mitogenome architectures are reported across the Tree of Life [38, 40]. For example, as previously mentioned, *P. robusta* also showed intraspecific architecture variability, with some mitochondrial structures containing introns, whereas others did not [48]. Our data on *P. ventilabrum* indicate the presence of two major mitochondrial lineages, primarily differentiated by an indel region of ~ 700 bp (Fig. 2). The cross-mapping strategy (mapping mitogroup “A” reads against the mitogroup “B” mitogenome, and vice versa) has been instrumental in validating by coverage that the mitogroup “A” assemblies were larger than initially inferred, as we found a coverage three times higher than the background coverage in the ~700 bp indel region (Fig. 2). These results suggest that the mitogroup “A” 683 bp indel could be the result of a misassembly produced by a repetitive region, possibly caused by a triple repetition from its homologous region. Regardless of whether the mitogenome “A” 683 bp indel reflects a misassembly, the differences observed between mitogenomes “A” and “B” point to underlying intraspecific variation. If the mitogenome “A” 683 bp indel is correctly assembled, its length is shorter relative to the mitogenome “B” due to the absence of the 683 bp region. Conversely, if the mitogenome “A” 683 bp indel is misassembled, it would likely contain three copies of the 683 bp region, which would further support the presence of structural variability between the two assemblies. A previous study on sponge mitogenomes demonstrated the abundance of Repetitive Hairpin-forming Elements (RHE) and their recurrent role in forming long intergenic regions [107]. The intraspecific variable region found in *P. ventilabrum* might be a product of the extreme mobility of RHE, followed by mutation in sponge mitogenomes, even within the same species. The cross-mapping strategy has also been previously used to validate the presence of insertions and deletions in the mitogenomes of an invasive ascidian species [38], highlighting indel regions spanning ~1,000 bp in the tunicate *Styela plicata* (Lesueur, 1823) [108]. We anticipate that in the near future, studies will be able to revisit unresolved mitogenome assemblies derived from Illumina WGS data by leveraging long-read sequencing technologies. Therefore, future studies should resolve the presence of hypothesized long indels and fully capture intraspecific variation in mitogenome architecture. In the meantime, the insightful results provided by this study encourage the cross-mapping assembly validation to be considered a state-of-the-art procedure in future intraspecific mitogenomic studies using Illumina WGS data.

Based on the mitochondrial structural variability of *P. ventilabrum*, our results suggest that mitogroups “B1” and “B2” are phylogenetically closer to each other than to “A”, as “B1” and “B2” share the same large indel (Fig. 2). This signal is concordant with the haplotype network reconstructed from whole mitogenome sequences, as the mitogroup “A” was the most differentiated structure (Fig. 3). Nevertheless, our phylogenetic inference analyses based on protein-coding genes and the *12S*, or removing the gappy and rearranged regions of the alignment (and therefore the aforementioned indels), fail to support a closer phylogenetic relationship between the two mitogroup “B” lineages (Fig. 4). However, it is important to mention that this phylogenetic relationship was not supported, and thus these results should be considered with caution. Unfortunately, software commonly used for phylogeny reconstruction has limitations dealing with insertions and deletions, hampering a proper phylogenetic evaluation when structural mutations are present [109]. Other explanations for node support recovery failures and mitochondrial gene tree inconsistencies have been reported across taxa. These include rapid radiations [110], which may preserve non-fixed polymorphisms among lineages. Also, gene-specific factors such as heterogeneous mutation loads related to the position relative to the origin of replication, as well as differences in the functional constraints acting on individual mitochondrial genes [111]. Therefore, these scenarios should not be ruled out. In any case, our results raise awareness about the limitations of relying solely on genic regions, as different evolutionary histories can be explained by micro- and macro-syntenic areas, thereby providing a more complex and comprehensive framework for the species of interest. Similar approaches have been key to demonstrating the evolutionary patterns of other taxa, such as birds, mammals, or ferns [112–114]. The combination of multiple genetic sources has historically been used in genetic studies by combining nuclear and mitochondrial protein-coding, intergenic, and rRNA molecular markers, as a good practice to avoid biases produced by the lack of diversity in data. Our study demonstrates that progressive synteny and genome tectonics, widely used in comparative genomic approaches with chromosome-level genome assemblies, should also be considered when evaluating the confidence of phylogenetic relationships among species or intraspecific individuals using mitochondrial data.

### Vicariant and admixed structure signals

Studies on genetic differentiation in demosponges using traditional mitochondrial markers have often been unsuccessful, as the commonly used *cox1* gene generally shows little to no variation among individuals in some sponge species [22, 27, 59, 61], or moderate resolution in others [62, 115, 116]. In fact, the low resolution of the F_ST_ analyses for each gene, combined with the fact that most genes showed few segregating sites and haplotypes, and extremely low nucleotide and haplotype diversity values, is in line with claims raised by previous research. The lack of mutations in sponge mitogenomes has been attributed to the presence of mitochondrial repair mechanisms, as also found in Cnidaria [58]. Unsurprisingly, when using the large first exon of the *cox1* of *P. ventilabrum*, we found that all specimens shared the same haplotype (Fig. 3), thus providing no resolution of genetic differentiation among the 28 specimens across the North Atlantic Ocean. Nevertheless, the *nad2* gene, with four mutations, recovered the same mitogroups as inferred by the whole mitogenome, although it could not differentiate further substructuring (Fig. 3). The large Tajima’s D values obtained for the *nad2* statistically support the hypothesis that this gene, despite harboring only four mutations, is effectively segregating the “A”, “B1”, and “B2” haplotypes. In demosponges, the DNA barcode *cox1* has one of the lowest mitochondrial substitution rates among metazoans [53]. Our finding is in agreement with the findings of Sperling and collaborators (2011) [117], who reported the absence of mutations in the *cox1* but variability in the *nad2* gene at the interspecific level (*Aplysina cauliformis* vs *Aplysina fulva*). Future studies should explore whether the *nad2* region is also variable in other sponge groups beyond *P. ventilabrum* and *Aplysina* sp.

For *P. ventilabrum,* whole mitogenomes provided the best resolution among the partitions tested here (Fig. 4), as their high-resolution level allowed for the differentiation of almost all individuals and proper lineage distinction within the species. This statement is supported by the highest number of differential indels and segregating sites, the highest F_ST_ across mitogroup pairwise comparisons, and elevated Tajima’s D. Our results support two main mitogroups differentiated by structural variants, the first encompassing the southern Cantabrian and Portuguese specimens (mitogroup “A”), and the other including all specimens from all northern localities (mitogroup “B”) (Fig. 4). Since the samples analyzed in this study spanned depths from 30 to 900 m, our results suggest that latitude, rather than habitat depth, is the primary driver of mitochondrial differentiation in *P. ventilabrum*. A similar Southern-Northern pattern was already recovered in a previous study on a larger set of *P. ventilabrum* specimens (from which we selected our samples) using ~4000 nuclear SNPs obtained through ddRADseq [19]. The authors suggested that oceanographic currents were mainly driving long-distance connectivity among the northernmost localities, but limiting the dispersal capabilities of specimens from the Cantabrian Sea [19]. The inclusion of new samples from a southernmost locality in Portugal and a northernmost locality in Iceland further corroborates their findings regarding the isolation between the southern and the northern genetic populations. Interestingly, mitogroup “A” specimens exhibited significantly lower mitoDNA-CN than mitogroup “B” samples. Variation in mitoDNA-CN has been associated with adaptive significance linked to energetic demand [118]. However, the functional role of mitoDNA-CN in *P. ventilabrum* should be tested in future studies.

Although Taboada et al., (2023), using the same specimens, described an almost panmictic population for the northern individuals they analyzed, we instead detected evidence of two independent mitochondrial lineages (“B1” and “B2”) co-occurring within the same sampling sites (Figs. 3, 4). Co-occurring independent mitochondrial lineages have been repeatedly proposed, in both marine (e.g. ascidians or seals) and terrestrial organisms (e.g. lizards or scorpions), to reflect ancient population splits followed by secondary contacts [38, 119–123]. Interestingly, a multi-taxon assessment of the North Atlantic benthos fauna indicated that post-Quaternary recolonization frequently led to secondary contact, since lineages isolated in glacial refugia (independent populations) expanded into the region and introgressed [124]. Our findings support the hypothesis that an ancient divergence led to two genetically distinct populations at both the nuclear and mitochondrial levels. Next, secondary contacts allowed interbreeding between previously isolated populations, promoting homogenization of the nuclear genome through recombination during meiosis and reshuffling of genetic material from both parents each generation. In contrast, the maternally-inherited non-recombining mitochondrial DNA was transmitted as a single genetic unit, thereby preventing its mixing across lineages. As a result, recurrent introgression may have reduced differentiation in nuclear markers, as observed by Taboada et al., (2023) using the same specimens, while allowing distinct mitochondrial haplotypes to persist (this study), preserving the signal of past divergence. In our case, all northern specimens share the same nuclear genomic information, while some specimens carry either the “B1” and the “B2” regardless of the sampling site. Thus, not only oceanographic currents would have influenced present-day phylogeographic patterns observed for *P. ventilabrum*, as already anticipated by Taboada et al. (2023) based on nuclear markers alone, but historical events may also have played a role in shaping the mitogenome signature. A possible hypothesis for the separation and subsequent admixture could involve quaternary glaciations, which are known to have left glacial refugia-related genetic signatures in various shallow-water benthic organisms due to the recurrent expansion and retraction of ice sheets and subsequent sea-level changes [23, 33, 124]. Future studies should aim to obtain a more extensive sampling and explore different demographic models to assess the Northern glacial refugium hypothesis.

It is worth mentioning that *P. ventilabrum* has a wide bathymetric range. Therefore, individuals of this species may have migrated to deeper habitats during glacial periods and the subsequent sea-level drops, which could have acted as thermal shelters. A similar biogeographic scenario has been recently suggested using demographic models based on nuclear data for the ground-forming sponge species *Geodia hentscheli* Cárdenas, Rapp, Schander & Tendal, 2010 [125], on which genetically distinct populations occurring at different bathymetric ranges have been detected, probably as a consequence of the rearrangement of prevalent oceanographic currents after the Last Glacial Maximum [23]. In *P. ventilabrum*, the clear genetic differentiation of the Iberian Peninsula samples at both nuclear and mitochondrial levels suggests that this area might also have been a latitudinal glacial refugium in the past, as it has already been suggested for brown algae [126] and the green crab *Carcinus maenas* (Linnaeus, 1758) [127, 128]. Possibly, both bathymetric and latitudinal hypotheses are correct, as multiple refugia originated during ancient glacial periods and subsequently, all of them connected and generated the present-day genetic structure. Future research should aim to reconcile mitochondrial and genomic data using extensive genomic data to elucidate the tempo and diversification of the different *P. ventilabrum* lineages through the reconstruction of complex demographic models, in order to fully resolve the entangled evolutionary history of this deep-sea ecologically important species. Previous, present, and future research on this species will lay the groundwork for science-based conservation and management actions in unique marine environments, such as North Atlantic sponge grounds, as the identification of independent and rare lineages can inform the design of marine protected areas.

## Conclusions

By combining whole-genome sequencing and RNA-seq data in an innovative way, we provide insights into mitogenome intron annotation using RNA-seq, confirm their mitochondrial intron splicing functionality, and establish a robust framework for validating atypical annotations in mitogenome studies. We expect this approach to be implemented in future studies to validate organellar introns in sponges and potentially discover new introns in other taxa across the Tree of Life. By using complete mitogenomes, we highlight their potential as effective tools for inferring maternally-inherited genomic differentiation in sponges. We highlighted that the mitogenome of *P. ventilabrum* hosts rich structural diversity, with three main lineages (“A”, “B1”, and “B2”) characterized by architectural mutations, each lineage fostering non-fixed insertions and deletions. However, due to the limitations of current Illumina sequencing, future studies leveraging long-read sequencing should aim to clarify the precise architecture of the 683 bp indel that distinguishes mitogroup “A” from mitogroups “B1” and “B2”. Finally, our *P. ventilabrum* mitochondrial data, combined with previously published nuclear data for the same specimens, together suggest a possible scenario of splits and secondary contacts, probably driven by glaciations and other environmental constraints. This study, in line with other studies, establishes mitogenomics as a powerful and accessible tool for assessing genomic differentiation and evolutionary trajectories in demosponges.

## Electronic supplementary material

Below is the link to the electronic supplementary material.

Supplementary Material 1

Supplementary Material 2

## Data Availability

All the raw data used in the present study have been deposited in the European Nucleotide Archive (ENA) under Bioproject number PRJEB100685. BioSample accession codes are available in Table 2. Mitogenome assemblies have been deposited in the NCBI, and their accession codes are available in Table 2. Mitogenome annotations can be found at GitHub (https://github.com/CGaliaCamps/Pventilabrum_mitogenomes)

## Abbreviations

*cox*: Cytochrome oxidase
*cob*: Cytochrome b
*nad*: Nicotinamide adenine dinucleotide
rRNA: Ribosomal RNA
tRNA: Transfer RNA
ufBS: Ultrafast bootstrap
