# Supplementary material for: Demosponge (Porifera) mitogenomes reflect latitudinal structuration, reveal intraspecific architecture variation, and confirm mitochondrial intron splicing
Source: Biol Direct. 2026 Jul 28;21:214. doi: 10.1186/s13062-026-00913-1 (PMC13700413; doi:10.1186/s13062-026-00913-1)
Supplement: Supplementary file 1 [file 13062_2026_913_MOESM1_ESM.docx]

**Demosponge (Porifera) mitogenomes reflect latitudinal structuration, reveal intraspecific architecture variation, and confirm mitochondrial intron splicing**

Carles-Galià-Camps^1,2,*^, Carlota Gracia Sancha^2^, Paco Cárdenas^3^, Joana R. Xavier^4,5^, Ana Riesgo^2^, Sergi Taboada^2^

**SUPPLEMENTARY FIGURES**

**Figure S1. Indel-rich alignments of the 28 specimens included in this study.** a) Alignment of a 1,032 bp region (mitogenome positions 7,923–8,955) enriched in short indels. b) Alignment of a 683 bp region containing a large indel (mitogenome positions 16,531–17,214).

**Figure S2: Visualization of exon–exon junctions in *P. ventilabrum* transcripts.** Reads supporting splicing partially map upstream of the intron 5′ boundary and downstream of the intron 3′ boundary, producing a split alignment separated by a gap corresponding to the excised intronic region (grey). Green and blue indicate forward and reverse reads, respectively, and red marks the split-read breakpoints. **a)** Junction reads spanning the *16S* intron. **b)** Junction reads spanning the *cox1* intron. Note that inner boxes are a zoom to correctly visualize split-read breakpoints. Read alignments were mapped against the Portugal_2 mitochondrial genome assembly.

**Figure S3: Mitochondrial copy number per mitogroup.** Above boxplots, asterisks show which statistical comparisons are significantly different based on t-tests and KS-tests.

**Figure S4: Phylogenetic trees and nodes support values** for the Supertree, Supermatrix and whole mitogenome inferences, independently.
